# Everolimus With Reduced Tacrolimus Improves Renal Function in *De Novo* Liver Transplant Recipients: A Randomized Controlled Trial

**DOI:** 10.1111/j.1600-6143.2012.04212.x

**Published:** 2012-11

**Authors:** P De Simone, F Nevens, L De Carlis, H J Metselaar, S Beckebaum, F Saliba, S Jonas, D Sudan, J Fung, L Fischer, C Duvoux, K D Chavin, B Koneru, M A Huang, W C Chapman, D Foltys, S Witte, H Jiang, J M Hexham, G Junge, for the H2304 Study Group

**Affiliations:** a General Surgery and Liver Transplantation, Azienda Ospedaliero-Universitaria PisanaPisa, Italy; b Department of Hepatology, University Hospital KU LeuvenLeuven, Belgium; c Hepato-biliary Surgery and Liver Transplantation Unit, Azienda Ospedaliera Niguarda Cà GrandaMilan, Italy; d Department of Gastroenterology & Hepatology, Erasmus MC, University Hospital Rotterdamthe Netherlands; e Department of General, Visceral and Transplantation Surgery, University Hospital Essen, Essen, Germany and Department of Transplant Medicine, University Hospital MünsterMünster, Germany; f Hepato-Biliary Center, AP-HP Hôpital Paul Brousse, Université Paris-Sud94804 Villejuif, France; g Department of Visceral, Transplantation, Thoracic and Vascular Surgery, University Medical Center LeipzigLeipzig, Germany; h Department of General Surgery, Division of Transplant Surgery, Duke University Medical CenterDurham, NC; i Transplantation Center, Cleveland ClinicCleveland, OH; j Department of Hepatobiliary Surgery and Transplantation, University Medical Center EppendorfHamburg, Germany; k Liver Transplant Unit, AP-HP Hôpital Henri MondorCréteil, France; l Division of Transplant Surgery, Medical University of South CarolinaCharleston, SC; m Department of Surgery, University of Medicine and Dentistry—New Jersey Medical SchoolNewark, NJ; n Division of Gastroenterology, Department of Internal Medicine, Henry Ford HospitalDetroit, MI; o Department of Surgery, Washington University School of MedicineSt. Louis, MO; p Department of Transplant Surgery, University Medical Center, Johannes Gutenberg UniversityMainz, Germany; q Novartis Pharma AGBasel, Switzerland; r Novartis PharmaceuticalsEast Hanover, NJ

**Keywords:** Efficacy, everolimus, liver transplantation, reduced, tacrolimus, withdrawal

## Abstract

In a prospective, multicenter, open-label study, *de novo* liver transplant patients were randomized at day 30±5 to (i) everolimus initiation with tacrolimus elimination (TAC Elimination) (ii) everolimus initiation with reduced-exposure tacrolimus (EVR+Reduced TAC) or (iii) standard-exposure tacrolimus (TAC Control). Randomization to TAC Elimination was terminated prematurely due to a higher rate of treated biopsy-proven acute rejection (tBPAR). EVR+Reduced TAC was noninferior to TAC Control for the primary efficacy endpoint (tBPAR, graft loss or death at 12 months posttransplantation): 6.7% versus 9.7% (−3.0%; 95% CI −8.7, 2.6%; p<0.001 for noninferiority [12% margin]). tBPAR occurred in 2.9% of EVR+Reduced TAC patients versus 7.0% of TAC Controls (p = 0.035). The change in adjusted estimated GFR from randomization to month 12 was superior with EVR+Reduced TAC versus TAC Control (difference 8.50 mL/min/1.73 m^2^, 97.5% CI 3.74, 13.27 mL/min/1.73 m^2^, p<0.001 for superiority). Drug discontinuation for adverse events occurred in 25.7% of EVR+Reduced TAC and 14.1% of TAC Controls (relative risk 1.82, 95% CI 1.25, 2.66). Relative risk of serious infections between the EVR+Reduced TAC group versus TAC Controls was 1.76 (95% CI 1.03, 3.00). Everolimus facilitates early tacrolimus minimization with comparable efficacy and superior renal function, compared to a standard tacrolimus exposure regimen 12 months after liver transplantation.

## Introduction

Calcineurin inhibitors (CNIs) remain the mainstay of immunosuppression following liver transplantation (1) but are associated with significant long-term complications including nephrotoxicity, which induces progressive, dose-related histological and functional renal deterioration (2,3). With more than 10% of liver transplant recipients progressing to severe chronic kidney disease by 5 years posttransplant (4,5), there is a pressing need to minimize CNI-related nephrotoxicity in the liver transplant population.

Immunosuppressants of the mammalian target of rapamycin (mTOR) inhibitor class act synergistically with CNIs (6), offering an opportunity to lower CNI exposure. However, evidence as to whether conversion from CNI- to mTOR inhibitor-based immunosuppression improves kidney function in patients with renal insufficiency after liver transplantation is conflicting (7–10), highlighting the need for early reduction or elimination of CNI exposure before irreversible renal deterioration has developed. No randomized trial has compared early introduction of everolimus combined with reduced CNI exposure to standard CNI therapy in a *de novo* liver transplant population.

This study was undertaken to evaluate the efficacy and safety of using everolimus to eliminate or reduce tacrolimus compared to a standard tacrolimus regimen in *de novo* liver transplant recipients.

## Methods

### Study design and conduct

A 24-month prospective, randomized, multicenter, three-arm, parallel-group and open-label study of adult *de novo* liver transplant recipients was undertaken at transplant centers in Europe, North/South America and Australia during the period from January 2008 to April 2011. The 12-month study period comprised a run-in period, with randomization performed 30 (±5) days posttransplant followed by an 11-month treatment period.

### Patients

Adult (18–70 years) recipients of a primary full-size liver transplant from a deceased donor, who had been initiated on an immunosuppressive regimen containing tacrolimus and corticosteroids (with or without mycophenolic acid [MPA]), were eligible to enter the run-in period. Key inclusion criteria for randomization comprised (i) acceptable graft function (aspartate aminotransferase [AST], alanine aminotransferase [ALT] and total bilirubin levels ≤3 times the upper limit of normal, with alkaline phosphatase ≤5 times the upper limit of normal), (ii) estimated GFR (eGFR) ≥30 mL/min/1.73 m^2^ (MDRD4) and (iii) tacrolimus trough concentration ≥8 ng/mL in the week prior to randomization. Key exclusion criteria included HCC that did not fulfill Milan criteria (11,12) at time of transplant as per explant histology, and receipt of antibody induction therapy. To enter the run-in period, patients were also excluded if urine protein to creatinine ratio indicated proteinuria (≥1.0 g/24 h). At the point of randomization (day 30), key additional inclusion criteria were Doppler ultrasound evidence showing the patency of hepatic artery, hepatic and portal veins; confirmation of eGFR ≥30 mL/min/1.73 m^2^; and the absence of acute rejection requiring antibody therapy or ≥1 episode of steroid-sensitive rejection during the run-in period.

### Randomization

Patients were stratified according to pretransplant hepatitis C (HCV) status (based on the presence/absence of anti-HCV antibodies) and quartiles of renal function at the time of randomization (based on eGFR [MDRD4]) in order to balance these risk factors for graft and patient survival, then randomized in a 1:1:1 ratio to (i) TAC Elimination, (ii) EVR+Reduced TAC, (iii) TAC Control.

In April 2010, the independent Data Monitoring Committee (DMC) recommended to stop the enrollment to the TAC Elimination arm due to a significantly higher rate of tBPAR in this group versus the other two treatment arms, which appeared to be clustered after tacrolimus withdrawal during days 120–180 after randomization. Randomization to the TAC Elimination arm was stopped, patients up to 180 days after randomization were converted to standard treatment, those who were more than 180 days postrandomization could continue on their assigned regimen, and a protocol amendment was implemented. At this point, approximately 690 patients had been randomized and eligible patients completing the run-in phase were randomized equally between the EVR+Reduced TAC and TAC Control groups.

### Intervention and concomitant medication

In the TAC Elimination arm, everolimus was initiated at a dose of 1.0 mg b.i.d. within 24 h of randomization with the dose adjusted from day 5 onward to maintain a trough (C_0_) concentration in the range 3–8 ng/mL until month 4 posttransplant, after which the target range increased to 6–10 ng/mL. Once everolimus trough concentration was in the range 3–8 ng/mL, tacrolimus dose was tapered to achieve tacrolimus trough concentration of 3–5 ng/mL by week 3 after randomization, then tacrolimus elimination was started after everolimus trough concentration achieved 6–10 ng/mL at the start of month 4 posttransplant and if liver function was confirmed to be adequate (see the “Patients” section). Tacrolimus elimination was to be completed by the end of month 4 after transplantation. In the EVR+Reduced TAC arm, everolimus therapy was initiated and monitored as for the TAC Elimination group, but the initial target range of 3–8 ng/mL was maintained throughout the study. Once everolimus trough concentration was within this range, tacrolimus dose was tapered to achieve a trough concentration by week 3 after randomization of 3–5 ng/mL, which remained unchanged for the remainder of the study. In the TAC Control arm, tacrolimus trough concentration was to be maintained in the range 8–12 ng/mL until month 4, after which the target range was 6–10 ng/mL until the end of the study.

For all patients, corticosteroids were to be initiated at the time of transplant and administered according to local practice (including perioperative intravenous corticosteroids), with a minimum oral dose of 5 mg prednisolone/day after randomization to be continued until at least month 6 posttransplant. MPA, if used, was administered as per local practice but had to be discontinued by the time of randomization.

### Study endpoints

The primary efficacy endpoint was the composite efficacy failure rate of treated biopsy-proven acute rejection (tBPAR), graft loss or death at 12 months posttransplantation (excluding events before randomization). tBPAR was defined as acute rejection with a locally confirmed rejection activity index (RAI) ≥3 according to Banff 1997 criteria (13) treated with antirejection therapy. All suspected cases of BPAR were to be assessed by biopsy and assessed locally. The key secondary endpoint was the change in renal function from randomization to month 12 posttransplant as assessed by estimated glomerular filtration rate (eGFR) using the four-variable modification of diet in renal disease (MDRD4) formula [14]). These endpoints were revised from the original endpoints after implementation of the protocol amendment to discontinue the TAC Elimination arm, in accordance with the EMA guideline on clinical investigation of immunosuppressants for solid organ transplantation (15). The original coprimary endpoints were noninferior composite efficacy failure rate of death, graft loss or loss to follow-up and superior renal function (as assessed by eGFR using the MDRD4 formula) at month 12 posttransplant.

The current analysis reports 12-month endpoints (intent-to-treat [ITT] population).

### Statistical analysis

A sample size of 242 patients per arm was calculated to provide (i) at least 80% power at the one-sided 0.0125 level for noninferiority of the EVR+Reduced TAC group versus the TAC Control arm in the proportion of patients with tBPAR, graft loss or death, assuming that both groups each have a true proportion of tBPAR, graft loss or death of 24% and a noninferiority margin of 12% (ii) at least 90% power at the one-sided 0.0125 level for noninferiority of the EVR+Reduced TAC group versus TAC Control for mean change in eGFR from randomization to month 12, assuming a noninferiority margin of the difference in eGFR is −6.0 mL/min with a standard deviation (SD) of 20 mL/min and a correlation coefficient with prerandomization eGFR of 0.5, using an analysis of covariance (ANCOVA) model.

Efficacy and renal function analyses were based on the ITT population, comprising all randomized patients. Safety analyses except renal function were based on the safety population, which included all randomized patients who received at least one dose of study medication.

## Results

Efficacy, renal function and safety data were reported for the EVR+Reduced TAC and TAC Control groups, but only limited safety data were presented for the TAC Elimination arm due to extensive conversion of patients from TAC Elimination to standard treatment after implementation of the DMC recommendation to stop randomization to this group. Statistical comparisons of the TAC Elimination group versus the other two treatment arms were not considered meaningful and are not shown.

### Patients

**Figure 1:**
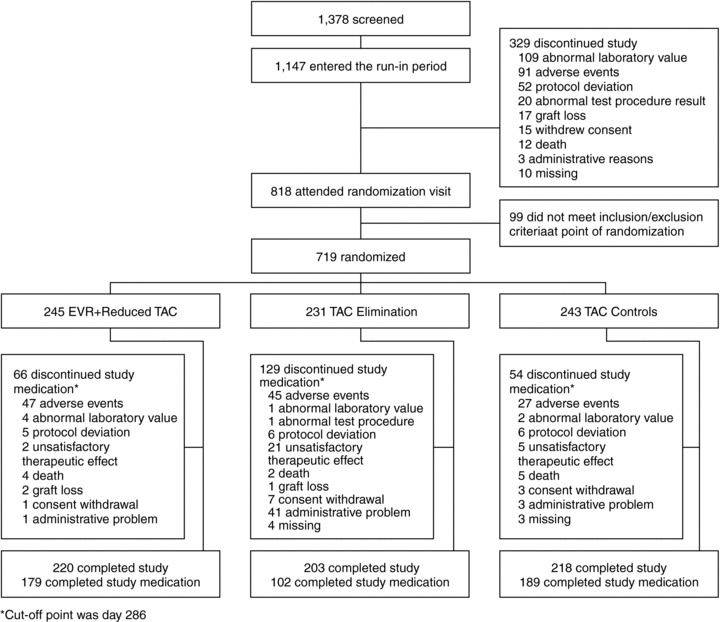
Patient disposition All randomized patients were included in the intent-to-treat (ITT) population (n = 719). The safety population excluded patients who were randomized but did not receive at least one dose of study medication (one TAC elimination patient and two TAC Control patients).

A total of 1147 patients underwent liver transplantation and entered the run-in period. Seven hundred and nineteen patients were eligible for randomization at day 30 and formed the ITT population (EVR+Reduced TAC 245, TAC Elimination 231, TAC Controls 243) (Figure 1). Three patients did not receive study medication (1 TAC Elimination, 2 TAC Controls), such that the safety population comprised 716 patients. The treatment groups were well balanced (Table 1).

**Table 1:** Demographics and baseline characteristics

	EVR+reduced TAC N = 245	TAC elimination N = 231	TAC controls N = 243
Age (years)	53.6 ± 9.2	53.2 ± 10.8	54.5 ± 8.7
Male gender, n (%)	180 (73.5)	164 (71.0)	179 (73.7)
Race, n (%)			
Caucasian	211 (86.1)	196 (84.8)	195 (80.2)
Black	4 (1.6)	6 (2.6)	9 (3.7)
Asian	4 (1.6)	8 (3.5)	5 (2.1)
Other	21 (8.2)	17 (7.4)	28 (0.7)
Missing	5 (2.0)	4 (1.7)	6 (2.5)
Body mass index (kg/m^2^)^1^	25.1 ± 4.2	25.3 ± 4.3	24.5 ± 4.2
HCV positive, n (%)	78 (31.8)	72 (31.2)	76 (31.3)
eGFR (MDRD4) (mL/min/1.73^2^)^1^	80.8 ± 32.7	82.9 ± 37.2	78.9 ± 27.7
Diabetic n (%)^1^	95 (38.8)	83 (35.9)	101 (41.6)
Primary disease leading to liver transplantation, n (%)			
Alcoholic cirrhosis	71 (29.0)	49 (21.2)	51 (21.0)
Hepatitis C	62 (25.3)	56 (24.2)	57 (23.5)
Hepatocellular carcinoma	42 (17.1)	31 (13.4)	35 (14.4)
Hepatitis B	17 (6.9)	17 (7.4)	15 (6.2)
Sclerosing cholangitis	8 (3.3)	20 (8.7)	12 (4.9)
Primary biliary cirrhosis	8 (3.3)	11 (4.8)	8 (3.3)
Metabolic disease	5 (2.0)	4 (1.7)	6 (2.5)
Cryptogenic cirrhosis	7 (2.9)	11 (4.8)	18 (7.4)
Autoimmune hepatitis	4 (1.6)	7 (3.0)	6 (2.5)
Acute hepatic failure	2 (0.8)	2 (0.9)	3 (1.2)
Other	19 (7.8)	23 (10.0)	32 (13.2)
MELD score^2^	19.2 ± 9.0	19.6 ± 7.5	19.0 ± 7.6
Donor age (years)	48.8 ± 18.2	50.0 ± 18.2	48.7 ± 17.4
Cold ischemia time (h)	8.4 ± 4.4	7.5 ± 2.7	8.0 ± 5.2
Acute rejection prior to randomization, n (%)			
tBPAR	15 (6.1)	10 (4.3)	13 (5.3)
BPAR	20 (8.2)	16 (6.9)	20 (8.2)
Acute rejection^3^	21 (8.6)	20 (8.7)	24 (9.9)

1 At randomization.

2 MELD score based on laboratory values only.

3 Clinically suspected acute rejection regardless of biopsy confirmation.

Continuous variables are shown as mean (SD).

BPAR = biopsy-proven acute rejection; tBPAR = treated biopsy-proven acute rejection; eGFR = estimated GFR; HCV = hepatitis C virus; MDRD4 = abbreviated modification of diet in renal disease; MELD = model for end-stage liver disease.

### Immunosuppression

At the time of randomization, 171 (70%), 151 (66%) and 168 (70%) patients in the EVR+Reduced TAC, TAC Elimination and TAC Control groups, respectively, were receiving mycophenolate mofetile, which was discontinued according to protocol.

At days 3–7 posttransplant, mean (SD) tacrolimus concentration was 6.1 (3.0) ng/mL and 6.0 (3.0) ng/mL in the EVR+Reduced TAC group and the TAC Control group, respectively; corresponding values at week 2 posttransplant were 8.5 (4.2) ng/mL and 8.9 (4.3) ng/mL. Supporting Figure S1 illustrates tacrolimus C_0_ concentrations from the time of randomization to month 12. In the EVR+Reduced TAC group, mean (SD) tacrolimus C_0_ concentration was 6.5 (5.2) ng/mL, 5.8 (5.8) ng/mL and 5.6 (6.3) ng/mL at months 3, 6 and 12 posttransplant, respectively, i.e. slightly above the target range. Corresponding values in the TAC Control group were 9.8 (3.2) ng/mL, 8.4 (3.8) ng/mL and 7.6 (2.8) ng/mL, all of which were within target range. The reduction in tacrolimus C_0_ concentration in the EVR+Reduced TAC group versus the TAC Control group varied from 26.3% to 38.4% at different timepoints during the study. Mean (SD) everolimus C_0_ concentration was within target ranges throughout the study in the EVR+Reduced TAC arm. After initial uptitration, the mean everolimus C_0_ concentration in the EVR+Reduced TAC group remained stable, within the range 5.5–6.3 ng/mL during months 3–12, with a maximum value observed at month 4.5 (6.3 [4.7] ng/mL). The mean (SD) dose of corticosteroids from randomization to month 12 was 0.15 (0.21) mg/kg/day in the TAC Elimination group, 0.20 (0.65) mg/kg/day in the EVR+Reduced TAC group and 0.13 (0.08) mg/kg/day in TAC Control.

The study was completed on-treatment to month 12 by 179 patients (73.1%) in the EVR+Reduced TAC group, 102 patients (44.2%) in the TAC Elimination group and 189 (77.8%) in the TAC Controls group (Figure 1).

### Efficacy

**Figure 2:**
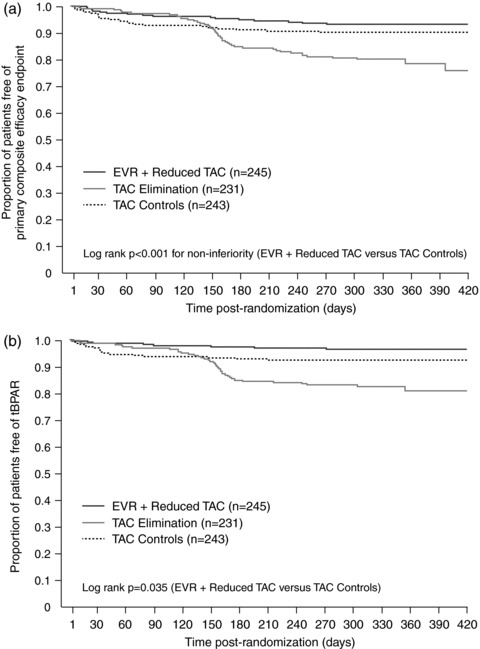
Kaplan–Meier plot for (a) the proportion of patients free from the primary composite efficacy endpoint of tBPAR, graft loss or death and (b) tBPAR (ITT population)

In the ITT population, the primary efficacy endpoint of tBPAR, graft loss or death at month 12 occurred in 45/231 patients (19.5%) in the TAC Elimination arm, 16/245 (6.5%) EVR+Reduced TAC subjects and 23/243 (9.5%) TAC Controls. To allow for censoring of the patients who were lost to follow-up, Kaplan–Meier incidence rates were calculated. The Kaplan–Meier incidence rate of the primary efficacy endpoint was statistically noninferior for EVR+Reduced TAC compared to TAC Controls: 6.7% versus 9.7%, respectively, with a difference of −3.0% (97.5% CI −8.7%, 2.6%) (p<0.001 for the noninferiority test with a noninferiority margin of 12%) (Table 2, Figure 2a). The incidence of graft loss or death, or either event individually, did not differ between the EVR+Reduced TAC group and TAC Controls, but the incidence of tBPAR (excluding events that occurred prior to randomization, i.e. tBPAR episodes between day 30 and month 12) was significantly lower in the EVR+Reduced TAC arm (Table 2, Figure 2b). No episodes of tBPAR in the EVR+Reduced TAC group were graded higher than RAI 4–5 (mild), compared to 9 episodes in the TAC Control group which were graded 6–7 (moderate) or 8–9 (severe) (Table 2). None of the graft losses in the EVR+Reduced TAC and TAC Control groups were related to rejection.

**Table 2:** Primary efficacy end point and selected secondary end points

				EVR+reduced TAC versus TAC controls		
				
	EVR+ reduced TAC N = 245	TAC elimination N = 231	TAC controls N = 243	Difference (97.5% CI)	Difference (95% CI)	p-Value
*Primary efficacy endpoint*^1^						
n	16	45	23	–	–	–
Kaplan–Meier incidence rate at month 12, %^2^	6.7	24.2	9.7	−3.0 (−8.7, 2.6)	–	<0.001
(noninferiority)^3^						
*Secondary end points (efficacy)*						
Graft loss, death, or loss to follow-up^4^	22 (9.0)	28 (12.1)	24 (9.9)	−0.9 (−7.3, 5.5)	–	<0.001
(noninferiority)^5^						
Graft loss or death, n (%)^7^	12 (4.9)	10 (4.3)	7 (2.9)	–	2.0 (−7.0, 10.9)	0.35
Graft loss, n (%)^7^	6 (2.4)	5 (2.2)	3 (1.2)	–	1.2 (−7.8, 10.2)	0.50
Death, n (%)^7^	9 (3.7)^7^	8 (3.5)^7^	6 (2.5)^7^	–	1.2 (−7.8, 10.1)	0.60
tBPAR, n (%)^8^	7 (2.9)	38 (16.5)	17 (7.0)	–	−4.1 (−8.0, −0.3)	0.035
RAI score (maximum severity)						
3	3 (1.2)	4 (1.7)	4 (1.6)			
4–5	4 (1.6)	16 (6.9)	4 (1.6)			
6–7	0	16 (6.9)	7 (2.9)			
8–9	0	2 (0.9)	2 (0.8)			
BPAR, n (%)^8^	10 (4.1)	46 (19.9)	26 (10.7)	–	−6.6 (−11.2, −2.0)	0.005
Acute rejection, n (%)^8^	9 (3.7)	46 (19.9)	26 (10.7)	–	−7.0 (−11.6, −2.5)	0.003
*Secondary end points (renal)*						
Change in eGFR from randomization to month 12 (MDRD4), mL/min/1.73 m^2^						
n	244	231	243		–	
Least squares mean (SE)	−2.23 (1.54)	−1.51 (1.58)	−10.73 (1.54)	−8.50 (3.74, 13.27)		<0.001 (superiority)^9^
						<0.001 (noninferiority)^10^
eGFR, mean (SD)^11^						
MDRD4, mL/min/1.73 m^2^ (14)						
Randomization	81.1 (32.6)	82.6 (37.2)	78.1 (27.5)	–	–	0.582
Month 12	80.9 (27.3)	80.8 (28.8)	70.3 (23.1)	–	–	<0.001
Cockcroft–Gault, mL/min (16)						
Randomization	87.3 (31.9)	88.6 (39.2)	81.1 (27.6)	–	–	0.060
Month 12	93.9 (36.3)	93.8 (38.5)	82.3 (32.0)	–	–	<0.001
Nankivell, mL/min/1.73 m^2^ (17)						
Randomization	91.2 (27.6)	91.6 (28.2)	87.2 (23.8)	–	–	0.301
Month 12	93.5 (23.4)	93.0 (24.4)	83.5 (21.5)	–	–	<0.001
CKD-EPI, mL/min/1.73 m^2^ (18)						
Randomization	78.2 (25.3)	78.6 (26.8)	76.6 (24.0)	–	–	0.481
Month 12	79.4 (24.1)	79.3 (25.6)	69.7 (20.8)	–	–	<0.001
Cystatin C, mL/min/1.73 m^2^ (19)						
Randomization						
Month 12	47.2 (14.4)	48.3 (14.6)	46.5 (12.7)	–	–	0.803
	59.1 (16.6)	58.9 (17.6)	54.5 (14.4)	–	–	0.005
Proteinuria, n (%)^6^						
Mean (SD)	0.25 (0.44)	0.24 (0.48)	0.16 (0.20)	–	–	0.003
≥0.5 g/day	26 (13.4)	19 (11.1)	11 (5.8)	–	–	0.015
≥1.0 g/day	8 (4.1)	10 (5.8)	2 (1.1)	–	–	0.105
≥3.0 g/day	1 (0.5)	1 (0.6)	0 (0.0)	–	–	1.000
Urine protein:creatinine ratio, mg/6						
Mean (SD)	246 (431)	237 (479)	151 (201)	–	–	0.008
≥30 (3.39 mg/mmol)	175 (100)	171 (100)	189 (100)	–	–	NA
≥500 (56.53 mg/mmol)	22 (11.3)	16 (9.4)	10 (5.3)	–	–	0.042
≥1000 (113 mg/mmol)	6 (3.1)	9 (5.3)	2 (1.1)	–	–	0.284
≥3000 (339 mg/mmol)	1 (0.5)	1 (0.6)	0	–	–	1.000

Local biopsy findings are shown. RAI = rejection activity index (11); eGFR = estimated GFR; MDRD4 = four-variable modification of diet in renal disease; CKD EPI = Chronic Kidney Disease Epidemiology Collaboration; NA = not applicable; HCV = hepatitis C virus; HCC = hepatocellular carcinoma.

1 Treated BPAR (tBPAR), graft loss or death.

2 Kaplan–Meier estimates.

3 Z-test (noninferiority margin 12%).

4 Initially planned primary efficacy endpoint.

5 Z-test (noninferiority margin 10%).

6 Two-sided Fisher exact test.

7 Deaths in EVR+reduced TAC group were due to multiorgan failure (2), acute hepatic failure, biliary sepsis, cardiac arrest, suicide (2), HCV and sepsis. Deaths in the TAC elimination group were due to HCV, cerebral hemorrhage (2), hepatic failure caused by recurrent HCV, histoplasmosis, recurrent HCC, operative hemorrhage and respiratory failure. Deaths in the TAC Control group were due to multiorgan failure (2), suicide, cardiac failure, chronic graft failure (biliary) and hepatic necrosis.

8 Pearson chi-square test (tBPAR occurring prior to randomization were excluded).

9 ANCOVA model, p value to test superiority.

10 Noninferiority margin -6mL/min/1.73m^2^, p value to test non-inferiority.

11 Wilcoxon rank sum test.

### Renal function

The change in adjusted eGFR (MDRD4) from randomization to month 12 was superior in the EVR+Reduced TAC group over TAC Control, with a difference of 8.50 mL/min/1.73 m^2^ (97.5% CI 3.74, 13.27 mL/min/1.73 m^2^, p < 0.001).

**Figure 3:**
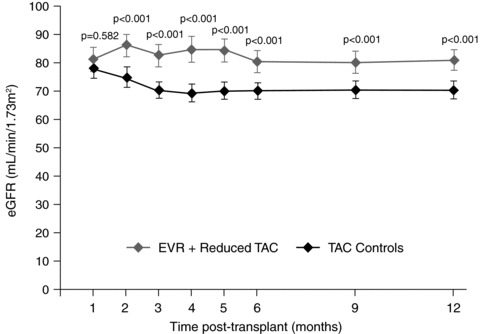
Estimated GFR (MDRD4) from randomization to month 12 posttransplant (ITT population) Values are shown as mean (SE).

A significant between-group difference in eGFR at month 12 was observed using MDRD4 and other formulae (Table 2). The difference in eGFR (MDRD4) values between the two groups was significant at all time points from week 6 posttransplant onward (all p < 0.001) (Figure 3). Urinary protein to creatinine ratio peaked at month 6 in the EVR+Reduced TAC group (median 105 mg/g, range 33–4143 mg/g), and at month 2 (median 108 mg/g, 39–10,370 mg/g) in the TAC Control arm. Mean values remained below 300 mg/g in both treatment arms at all time points. None of the nine patients in the EVR+Reduced TAC group who had a preexisting urinary protein to creatinine ratio ≥500 mg/g (but lower than 1.0 g/24 h) showed an increase at month 12. Renal replacement therapy was required in six, three and four patients in the EVR+Reduced TAC, TAC Elimination and TAC Control arms, respectively. No patient remained on renal replacement therapy at month 12. Of these, three, two and four cases, respectively, occurred in patients who were in critical care and subsequently died.

### Safety

The proportion of patients experiencing one or more adverse event, or serious adverse event, was similar between the EVR+Reduced TAC group and the TAC Control arm (Table 3). The incidence of individual adverse events did not differ between the two groups other than a higher risk of peripheral edema and leukopenia in the EVR+Reduced TAC patients. The incidence of leukopenia, thrombocytopenia and anemia in the EVR+Reduced TAC patients was 11.8%, 5.3% and 7.8%, respectively, compared to 5.0%, 1.7% and 8.3% in the TAC Controls. Interstitial lung disease was reported for one patient in each of the three treatment groups.

**Table 3:** Adverse events and serious adverse events, n (%)

	EVR+ reduced TAC N = 245	TAC elimination N = 230	TAC controls N = 241	Relative risk (95% CI)^a^
Any adverse event	232 (94.7)	216 (93.9)	229 (95.0)	1.00 (0.96, 1.04)
Any serious adverse event	122 (49.8)	130 (56.5)	104 (43.2)	1.15 (0.95, 1.40)
Any infection	123 (50.2)	114 (49.6)	105 (43.6)	1.15 (0.95, 1.39)
Any serious infection	34 (13.9)	49 (21.3)	19 (7.9)	1.76 (1.03, 3.00)
Adverse events leading to study drug discontinuation	63 (25.7)	60 (26.1)	34 (14.1)	1.82 (1.25, 2.66)
Adverse events occurring in ≥10% of patients in any group				
Diarrhea	47 (19.2)	54 (23.5)	50 (20.7)	0.92 (0.65, 1.32)
Headache	47 (19.2)	39 (17.0)	46 (19.1)	1.01 (0.70, 1.45)
Peripheral edema	43 (17.6)	42 (18.3)	26 (10.8)	1.63 (1.03, 2.56)
Hypertension	42 (17.1)	32 (13.9)	38 (15.8)	1.09 (0.73, 1.62)
Nausea	33 (13.5)	24 (10.4)	28 (11.6)	1.16 (0.72, 1.86)
Abdominal pain	32 (13.1)	29 (12.6)	22 (9.1)	1.43 (0.86, 2.39)
Pyrexia	32 (13.1)	45 (19.6)	25 (10.4)	1.26 (0.77, 2.06)
Leukopenia	29 (11.8)	21 (9.1)	12 (5.0)	2.38 (1.24, 4.55)
Hepatitis C	28 (11.4)	21 (9.1)	19 (7.9)	1.45 (0.83, 2.52)
Tremor	23 (9.4)	17 (7.4)	29 (12.0)	0.78 (0.46, 1.31)
Fatigue	22 (9.0)	20 (8.7)	26 (10.8)	0.83 (0.49, 1.43)
Anemia	19 (7.8)	25 (10.9)	20 (8.3)	0.93 (0.51, 1.71)
Serious adverse events occurring in ≥2% of patients in any group				
Pyrexia	10 (4.1)	16 (7.0)	6 (2.5)	1.64 (0.61, 4.44)
Cholangitis	9 (3.7)	8 (3.5)	5 (2.1)	1.77 (0.60, 5.21)
Hepatitis C	8 (3.3)	7 (3.0)	3 (1.2)	2.62 (0.70, 9.77)
Acute renal failure	8 (3.3)	3 (1.3)	1 (0.4)	7.87 (0.99, 62.44)
Incisional hernia	7 (2.9)	2 (0.9)	3 (1.2)	2.30 (0.60, 8.77)
Cholestasis	5 (2.0)	1 (0.4)	2 (0.8)	2.46 (0.48, 12.55)
Sepsis	5 (2.0)	3 (1.3)	3 (1.2)	1.64 (0.40, 6.78)
Renal failure	5 (2.0)	4 (1.7)	3 (1.2)	1.64 (0.40, 6.78)
Abdominal hernia	4 (1.6)	8 (3.5)	4 (1.7)	0.98 (0.25, 3.89)
Bile duct stenosis	4 (1.6)	5 (2.2)	4 (1.7)	0.98 (0.25, 3.89)
Diarrhea	3 (1.2)	9 (3.9)	2 (0.8)	1.48 (0.25, 8.75)
Pneumonia	3 (1.2)	8 (3.5)	4 (1.7)	0.74 (0.17, 3.26)
Biliary anastomosis complication	2 (0.8)	3 (1.3)	7 (2.9)	0.28 (0.06, 1.34)
Graft rejection	1 (0.4)	15 (6.5)	3 (1.2)	0.33 (0.03, 3.13)
Adverse events leading to study drug discontinuation in ≥1% of patients in any group				
Proteinuria	7 (2.9)	2 (0.9)	1 (0.4)	6.89 (0.85, 55.54)
Hepatitis C	5 (2.0)	5 (2.2)	3 (1.2)	1.64 (0.40, 6.78)
Graft loss	3 (1.2)	0 (0.0)	0 (0.0)	NA
Pancytopenia	3 (1.2)	1 (0.4)	0 (0.0)	NA
Leukopenia	1 (0.4)	4 (1.7)	0 (0.0)	NA
Thrombocytopenia	1 (0.4)	4 (1.7)	0 (0.0)	NA
Graft rejection	0 (0.0)	10 (4.3)	1 (0.4)	NA
Renal failure	0 (0.0)	1 (0.4)	4 (1.7)	NA

a EVR+Reduced TAC versus TAC controls.

During the randomized treatment period, hepatic artery thrombosis (HAT) was reported for one EVR+Reduced TAC patient. This was a second episode of HAT in the same patient, with the first having occurred during the run-in period, requiring reanastomosis of the hepatic artery and stent placement. A late and temporary hepatic artery occlusion without graft loss was reported for one TAC Elimination patient that resolved under heparin. This compares to 14 patients with HAT during the prerandomization run-in phase.

Wound healing complications were reported in a similar proportion of patients in each group: 11.0% (n = 27), 9.6% (n = 22) and 7.9% (n = 19) of the EVR+Reduced TAC, TAC Elimination and TAC Control patients (RR 1.40, 95% CI 0.80, 2.45 between EVR+Reduced TAC and TAC Control groups).

The overall incidence of infections was similar between groups (Table 3), as was the incidence of viral infections (17.1%[n = 42] of EVR+Reduced TAC patients, 13.3%[n = 32] of TAC Controls (RR 1.29, 95% CI 0.84, 1.97)). Cytomegalovirus (CMV) viremia was detected at a similar rate between groups (EVR+Reduced TAC 6.5%, TAC Controls 6.6%). The relative risk of serious infections in the EVR+Reduced TAC group versus TAC Controls was 1.76 (95% CI 1.03, 3.00). The incidence of pneumonia as a serious adverse event was similar in the EVR+Reduced TAC and TAC Control arms (three and four patients, respectively). HCV was reported as a serious infection (according to investigators’ judgment) in eight patients in the EVR+Reduced TAC group (3.3%) and three TAC Control patients (1.2%). HCV viral load was imbalanced between groups at screening (log_10_ transformed mean: 5.9 for the EVR+Reduced group versus 5.6 for the TAC Control group) which was maintained at month 12 (6.6 and 6.1, respectively). The change in HCV viral load was similar in both groups (log_10_ transformed mean: 0.54 for the EVR+Reduced TAC group versus 0.51 for the TAC Control group).

Neoplasms were reported in 10 EVR+Reduced TAC patients (4.1%) and 16 TAC Control patients (6.6%) (RR 0.61, 95% CI 0.28, 1.33). Recurrence of HCC occurred in one patient each in EVR+Reduced TAC (bone metastasis) and TAC Elimination (peritoneal metastases), both of whom subsequently died from preexisting metastatic and progressive extrahepatic HCC.

Discontinuation of study drug due to adverse events occurred in more patients in the EVR+Reduced TAC arm (n = 63, 25.7%) compared to TAC Controls (n = 34, 14.1%) by the end of month 12 (Table 3). Four patients experienced wound-healing events that were associated with study drug discontinuation (cellulitis and wound erythema in two patients in the EVR+Reduced TAC group, delayed healing in a patient in the TAC Elimination group and wound infection in a TAC Control patient).

Lipid-lowering treatment was administered to 23.3% (n = 57) of patients in the EVR+Reduced TAC group and 17.8% (n = 43) in TAC Controls (p = 0.944), most frequently therapy with statins. In EVR+Reduced TAC versus TAC Control patients, mean (SD) values at month 12 were 209 (43) mg/dL versus 175 (44) mg/dL for total cholesterol (p < 0.001), 121 (35) mg/dL versus 101 (34) mg/dL for LDL-cholesterol (p < 0.001), 51 (17) mg/dL versus 47 (15) mg/dL for HDL-cholesterol (p = 0.029), and 197 (136) mg/dL versus 141(78) mg/dL for triglycerides (p < 0.001), respectively. The incidence of new onset diabetes mellitus (defined as two consecutive fasting plasma glucose >126 mg/dL [7.0 mmol/L] >30 days posttransplant, HbA1c >6.5% from day 75 onward, diabetes reported as an adverse event after day 30, or use of antidiabetic medication after day 30 for >30 days) among patients nondiabetic at randomization was 32.0% (n = 48/150) in the EVR+Reduced TAC group and 28.6% (n = 40/140) in the TAC Control arm (p = 0.609).

## Discussion

Identifying an immunosuppressive regimen that preserves renal function while maintaining efficacy represents an urgent unmet medical need after liver transplantation (20). The need for such a regimen has become particularly pressing as the proportion of transplanted patients with renal insufficiency has increased during the model for end-stage liver disease (MELD) era (21). In this, the largest registration trial ever undertaken in liver transplantation, EVR+Reduced TAC demonstrated superior renal function to the TAC Control arm at 1 year posttransplant, with a difference that was clinically relevant, in standard-risk patients without compromising efficacy and with an acceptable safety profile.

During the first year after transplantation, mean eGFR remained close to baseline levels in the EVR+Reduced TAC arm but declined in the TAC Control arm, reaching a significant difference of 8.50 mL/min/1.73 m^2^ in change of eGFR from randomization (month 1) to month 12. Superiority in the EVR+Reduced TAC group was apparent as early as 1 month after introduction of everolimus (Figure 3), with no apparent decline over time. GFR at 1 year after liver transplantation has consistently been shown to be an independent predictor of progressive renal deterioration and kidney function at 5 years (22–24), so the benefits of the EVR+Reduced TAC regimen in terms of maintaining renal function may be maintained over the long term. The between-group difference in renal function was confirmed by all the formulae that were used. The size of the renal benefit seen here, (8.50 mL/min/1.73 m^2^), in a population with mean renal function close to normal, contrasts with the clinically less relevant difference of 3.38 mL/min observed in a recent metaanalysis of studies in which liver transplant patients were converted to a sirolimus-based regimen only after renal insufficiency had developed (9).

Despite a substantial reduction in tacrolimus exposure, the EVR+Reduced TAC arm was statistically noninferior to the control arm for the primary efficacy endpoint of tBPAR, graft loss or death. The incidences of tBPAR and BPAR were significantly lower in the EVR+Reduced TAC cohort, and no moderate or severe episodes of rejection were reported with this regimen (tBPAR or BPAR occurring prior to randomization were excluded). The difference became apparent during the first 2 months postrandomization, when the risk of cellular rejection is high. It should be noted that the tacrolimus C_0_ concentration in the EVR+Reduced TAC group was in the range of 5–6 ng/mL, slightly above the upper extent of the target range (3–5 ng/mL) throughout the study, which may have influenced the results, and suggests that somewhat lower tacrolimus exposure could be explored to further reduce CNI-related nephrotoxicity. The patient and graft loss were not significantly different between the EVR+Reduced TAC and control arms, and there were no everolimus-related deaths (Table 2).

The TAC Elimination group was terminated prematurely based on a recommendation of the DMC due to a higher incidence of acute rejection versus the other cohorts (there were no concerns relating to graft loss or mortality). The episodes of rejection were clustered around the time when tacrolimus was eliminated. During development of the study protocol, it was thought that lowering the overall intensity of immunosuppression by switching to a CNI-free regimen 4 months after transplantation might be feasible in liver transplant recipients since most acute rejections occur during the first 8–12 weeks posttransplant. However, results showed that mTOR inhibition without induction therapy and without additional immunosuppressive comedication (e.g. IMPDH inhibition) is not feasible as early as 90 days posttransplant in a largely unselected liver transplant population. In contrast, an acceptable level of rejection has been achieved using an everolimus-based regimen with basiliximab induction in which tacrolimus was withdrawn stepwise over a period of 8 weeks, an approach that merits further investigation (25). In this study, the statistical analysis did not include the TAC Elimination arm since all but 90 of patients randomized to TAC Elimination were converted to local standard treatment, such that an ITT analysis was not considered valid. The incidence of treated BPAR in the TAC Elimination group (16.5%) was still comparable to other randomized controlled studies (26–28) and most episodes were mild or moderate, with less than 1% of patients in the TAC Elimination arm experiencing severe rejection. However, given the variation in treatment within the group no robust conclusions can be drawn.

Safety results were consistent with the known class effects of mTOR inhibitors. Approximately three-quarters of patients tolerated the EVR+Reduced TAC regimen to month 12. There were only minor differences in the occurrence of wound healing complications between groups. Lipid levels were slightly increased in the EVR+Reduced TAC group versus Controls, but remained in the upper range of normal or were borderline elevated. The incidence of anemia was similar in both groups. There was a trend toward a higher rate of serious infections in the EVR+Reduced TAC group, but no difference in pneumonia as a serious adverse event. Proteinuria was observed in the EVR+Reduced TAC group, but maximum mean values for urinary protein to creatinine ratio were below even the conservative threshold of 300 mg/g, and preexisting cases of proteinuria were not aggravated. No patient developed severe renal dysfunction (eGFR <30 mL/min/1.73 m^2^). The tubular damage and consequent proteinuria that have been reported by some centers using mTOR inhibitors in kidney transplantation (29) may be of less concern in liver transplant recipients.

Certain aspects of the study merit consideration. The timing of everolimus introduction (day 30) was selected to ensure CNI reduction before irreversible kidney damage occurs (7) and also to minimize the risks of wound-healing complications (30,31) and potential vascular complications such as HAT (32,33). By delaying introduction of everolimus until day 30, these aims were achieved since the rates of such events were low and comparable between groups. Future studies may reveal whether earlier introduction of everolimus (e.g. 10–15 days after transplantation) can achieve more pronounced renal benefits while maintaining safety and tolerability. Key limitations of the trial are as follows. First, the target ranges for tacrolimus exposure, established during protocol development in 2007, may be higher than may now be standard. More modern regimens using lower tacrolimus trough concentrations in everolimus-treated liver transplant patients may achieve a more pronounced renal benefit. Second, use of an open-label design, which was necessary due to the need for sensitive adjustments of tacrolimus and everolimus exposure, may have biased adverse event reporting (most other endpoints were nonsubjective). Third, randomized patients had a mean eGFR of ∼80 mL/min/1.73 m^2^, i.e. close to normal, even though the inclusion criteria stipulated eGFR ≥30 mL/min/1.73 m^2^. This means that the deterioration in renal function in the control arm can confidently be attributed to CNI exposure. Categorical shift analyses by renal function strata demonstrated that a greater proportion of patients with baseline eGFR in the range 30 to <45 mL/min/1.73 m^2^ or 45 to <60 mL/min/1.73 m^2^ shifted to a higher category of eGFR by month 12 when treated with EVR+Reduced TAC compared to Controls (data not shown), suggesting that the overall results are also likely to apply to patients with renal function in the range 30–60 mL/min/1.73 m^2^ at time of liver transplantation. Fourth, the control arm did not include MPA, which may have affected efficacy outcomes. Although coadministration of MPA with TAC is not approved by the Food and Drug Administration for this indication, it is now frequently used in liver transplant patients. Fifth, kidney function was estimated instead of being measured directly. However, multiple methods of estimating GFR were used to improve accuracy, including the new CKD-EPI (18) and cystatin C (19) equations, each of which confirmed a similar pattern of superior renal function with EVR+Reduced TAC. Lastly, the results reported here extend to only 1 year posttransplant, although full 2-year results will follow. Longer follow-up may permit an assessment of other potential advantages of an mTOR inhibitor-based regimen, notably decreased recurrence of HCC (34,35) and HCV, which remain urgent unmet medical needs in liver transplantation.

In conclusion, introduction of everolimus with tacrolimus reduction from day 30 achieved superior renal function with no compromise in efficacy at 12 months after liver transplantation. The safety profile of EVR+Reduced TAC presented no unexpected safety concerns and showed similar tolerability to the standard tacrolimus regimen. Thus, early initiation of everolimus at a targeted blood trough level of 3–8 ng/mL in combination with reduced exposure tacrolimus provides a positive outcome in terms of benefit to risk ratio, representing a valid alternative to existing regimens in liver transplantation.

## Appendix: The H2304 Study Investigators

*Argentina:* A.C. Gadano, Buenos Aires; L. McCormack, Buenos Aires; L. Toselli, San Martin; S. Yantorno, Buenos Aires; F. Zingalie, Rosario. *Australia:* J. Chen, Bedford Park; G. Jeffrey, Nedlands; R. Jones, Heidelberg; G. McCaughan, Camperdown. *Brazil:* G. Cantisani, Porto Alegre; O. Castro, Ribeirao Preto; L.F. Moreira, Rio de Janerio; J.C. Wiederkehr, Blumenau. *Belgium:* O. Detry, Liege; F. Nevens, Leuven; X. Rogiers, Gent. *Canada:* N. Kneteman, Edmonton; P. Marotta, London; G. Therapondros, Toronto; E. Yoshida, Vancouver. *Colombia:* L. Caicedo, Cali; G. Mejía, Bogotá. *Czech Republic*: P. Trunecka, Praha. *France:* E. Boleslawski, Lille; F. Durand, Clichy; C. Duvoux, Creteil; J. Hardwigsen, Marseilles; M. Neau-Cransac, Bordeaux; G. Pageaux, Montpellier; F. Saliba, Villejuif. *Germany:* L. Fischer, Hamburg; D.B. Foltys, Mainz; S. Jonas, Leipzig; S. Beckebaum, Essen; P. Neuhaus, Berlin; J. Schmidt, Heidelberg; A. Schnitzbauer, Regensburg. *Hungary:* J. Jaray, Budapest. *Ireland:* A. McCormick, Dublin. *Israel:* R. Nakache, Tel-Aviv. *Italy:* L. De Carlis, Milan; F. Filopponi, Pisa; G.E. Gerunda, Modena; M. Rossi, Rome; M. Salizzoni, Turin. *Netherlands:* H.J. Metselaar, Rotterdam. *Russia:* S. Gautier, Moscow; A.V. Zhao, Moscow. *Spain:* B. Bilbao Aguirre, Barcelona; J. Fabregat, Llobregat; I. Herero, Pamplona; V. Cuervas Mons, Majadanonda; A. Moya, Valencia; M. Navasa, Barcelona; J. Ortiz de Urbina Baracaldo; M. Salcedo, Madrid. *Sweden:* B-G Ericzon, Stockholm. *United Kingdom:* N. Heaton, London; A. MacGilchrist, Edinburgh. *USA:* A. Alsina, Tampa; R. Brown, New York; S.Gordon Burroughs, Houston; W.C. Chapman, St Louis; K. Chavin, Charleston; S.S. Cheng, Dallas; S.D. Coloquhoun, Los Angeles; C. Doria, Philadelphia; G. Everson, Aurora; S. Feng, San Francisco; J.J. Fung, Cleveland; R. Gedaly, Lexington; S. Geevarghese, Nashville; J. Goss, Houston; J. Hong, Los Angeles; M.A. Huang, Detroit; L. Johnson, Washington; M. Johnson, Atlanta; G.B. Klintmalm, Dallas; V. Kohli, Oklahoma City; B. Koneru, Newark; T. Kozlowski, Chapel Hill; H Merhav, Houston; S. Orloff, Portland; L. Sher, Los Angeles; D. Sudan, Durham; L.W. Teperman, New York; G. Testa, Chicago; K. Watt, Rochester.

## Abbreviations

ALT: alanine aminotransferase
ANCOVA: analysis of covariance
AST: aspartate aminotransferase
BPAR: biopsy-proven acute rejection
CKD EPI: Chronic Kidney Disease Epidemiology Collaboration
CMV: cytomegalovirus
CNI: calcineurin inhibitor
DMC: Data Monitoring Committee
EGFR: estimated glomerular filtration rate
HAT: hepatic artery thrombosis
HCC: hepatocellular carcinoma
HCV: hepatitis C virus
ITT: intent-to-treat
MDRD: modification of diet in renal disease
MELD: model for end-stage liver disease
MPA: mycophenolic acid
MTOR: mammalian target of rapamycin
RAI: rejection activity index
RR: relative risk
SD: standard deviation
SE: standard error
TBPAR: treated biopsy-proven acute rejection

## Supporting Information

**Supporting Methods:** Study design and conduct, patients, randomization, intervention and concomitant medication, study endpoints, statistical analysis.

**Figure S1:** Tacrolimus trough concentration from randomization to month 12 posttransplant (safety population). Values are shown as mean (SD).
