# Supplementary material for: Everolimus With Reduced Tacrolimus Improves Renal Function in De Novo Liver Transplant Recipients: A Randomized Controlled Trial
Source: Am J Transplant. 2012 Nov;12(11):3008–20. doi: 10.1111/j.1600-6143.2012.04212.x (PMC3533764; doi:10.1111/j.1600-6143.2012.04212.x)
Supplement: Supplementary file 1 [file ajt0012-3008-SD1.doc]

**Everolimus with reduced tacrolimus improves renal function in *de novo* liver transplant recipients: a randomized controlled trial**

De Simone P et al

*Study design and conduct*

The trial was initiated and funded by Novartis Pharma AG. Participating centers provided data electronically to a Novartis database. The study protocol was developed by Novartis in collaboration with a Scientific Steering Committee. Novartis undertook monitoring of study conduct, data quality control and statistical analysis. The Scientific Steering Committee, which included the lead author, had full access to the study data and made the decision to publish, and vouches for the veracity and completeness of the data and the data analysis. All authors reviewed and commented on drafts of the manuscript.

The trial was conducted in accordance with the Declaration of Helsinki and Good Clinical Practice guidelines. All patients provided written informed consent.

*Patients*

Additional exclusion criteria included receipt of a graft from a living donor or a split liver, a multiorgan transplant, combined liver transplant, or any previous organ or tissue transplantation; receipt of an ABO incompatible graft or from a donor who tested positive for hepatitis B surface antigen or HIV.

*Randomization*

Randomization was performed using a validated, automated system and investigators were informed of the allocated randomization group via an interactive voice response system. In this open-label trial, the investigator, pharmacist and patient were aware of the randomization group, but data analysts remained blinded until database lock after all patients completed the first 12 months of the study.

Patients randomized to the TAC Elimination arm, who were converted to local standard treatment, and patients in any treatment group who discontinued study medication were converted to local standard treatment, were followed to the end of the study.

*Intervention and concomitant medication*

Everolimus dose changes were made based on everolimus C_0_ concentrations measured by liquid chromatography mass spectrometry (LCMS). Tacrolimus dose adjustments were based on local measurements of C_0_ concentration.

The occurrence of adverse events was sought by non-directive questioning of the patient at each study visit (baseline, weeks 2, 4, 5 and 6, and months 2, 4, 5, 6, 9 and 12). Adverse events could also be detected when volunteered by the patient during or between visits or through physical examination, laboratory test, or other assessments.

*Study endpoints*

The occurrence of adverse events was sought by non-directive questioning of the patient at each study visit (baseline, weeks 2, 4, 5 and 6, and months 2, 4, 5, 6, 9 and 12). Adverse events could also be detected when volunteered by the patient during or between visits or through physical examination, laboratory test, or other assessments.

Additional secondary efficacy endpoints at month 12 included separate incidences of components of the composite efficacy endpoint, and the severity of tBPAR as assessed by the local pathologist according to Banff 1997 criteria (1). Safety endpoints at month 12 included the premature discontinuation of study medication, the incidence of adverse events, severe adverse events, incidence of new-onset diabetes, hepatocellular carcinoma (HCC) recurrence, laboratory values and vital signs.

*Statistical analysis*

The primary endpoint of composite efficacy failure at month 12 was analyzed by calculating two-sided 97.5% confidence intervals (CI) based on the Z-test for the between-group difference in incidence using a pre-specified 12% non-inferiority margin. Originally, Hochberg’s procedure was planned to ensure the overall Type I error rate not to be over 5% for testing both TAC Elimination and EVR+Reduced TAC regimens compared to the TAC Controls. Due to the stopping of the TAC Elimination arm, a Type I error rate of two-sided 2.5% (one-sided 1.25%, two-sided 97.5% confidence interval) was used for the hypothesis testing of the EVR+Reduced TAC exposure regimen versus the TAC Controls.

The incidence of the composite efficacy failure endpoint was estimated using the Kaplan-Meier product-limit formula and standard error (SE) based on Greenwood’s formula*.*

The key secondary endpoint of change in eGFR from randomization to month 12 post-transplant was analyzed using an ANCOVA model, with the change in eGFR (MDRD4) from randomization to month 12 representing the response variable and treatment group, pre-transplant HCV status and eGFR (MDRD4) at randomization as covariates, using two-sided 97.5% CI and a non-inferiority margin of -6mL/min/1.73m^2^. Relative risk (risk ratio)(RR) values and the corresponding 95% confidence intervals (CI) between the EVR+Reduced TAC and TAC Control groups were calculated for safety endpoints including overall adverse events, serious adverse events, infections and serious infection and selected adverse events of interest.

**Reference**1. Banff schema for grading liver allograft rejection: an international consensus document. Hepatology 1997;25:658-663.
