# Supplementary figures and images for: Everolimus With Reduced Tacrolimus Improves Renal Function in De Novo Liver Transplant Recipients: A Randomized Controlled Trial
Source: Am J Transplant. 2012 Nov;12(11):3008–20. doi: 10.1111/j.1600-6143.2012.04212.x (PMC3533764; doi:10.1111/j.1600-6143.2012.04212.x)

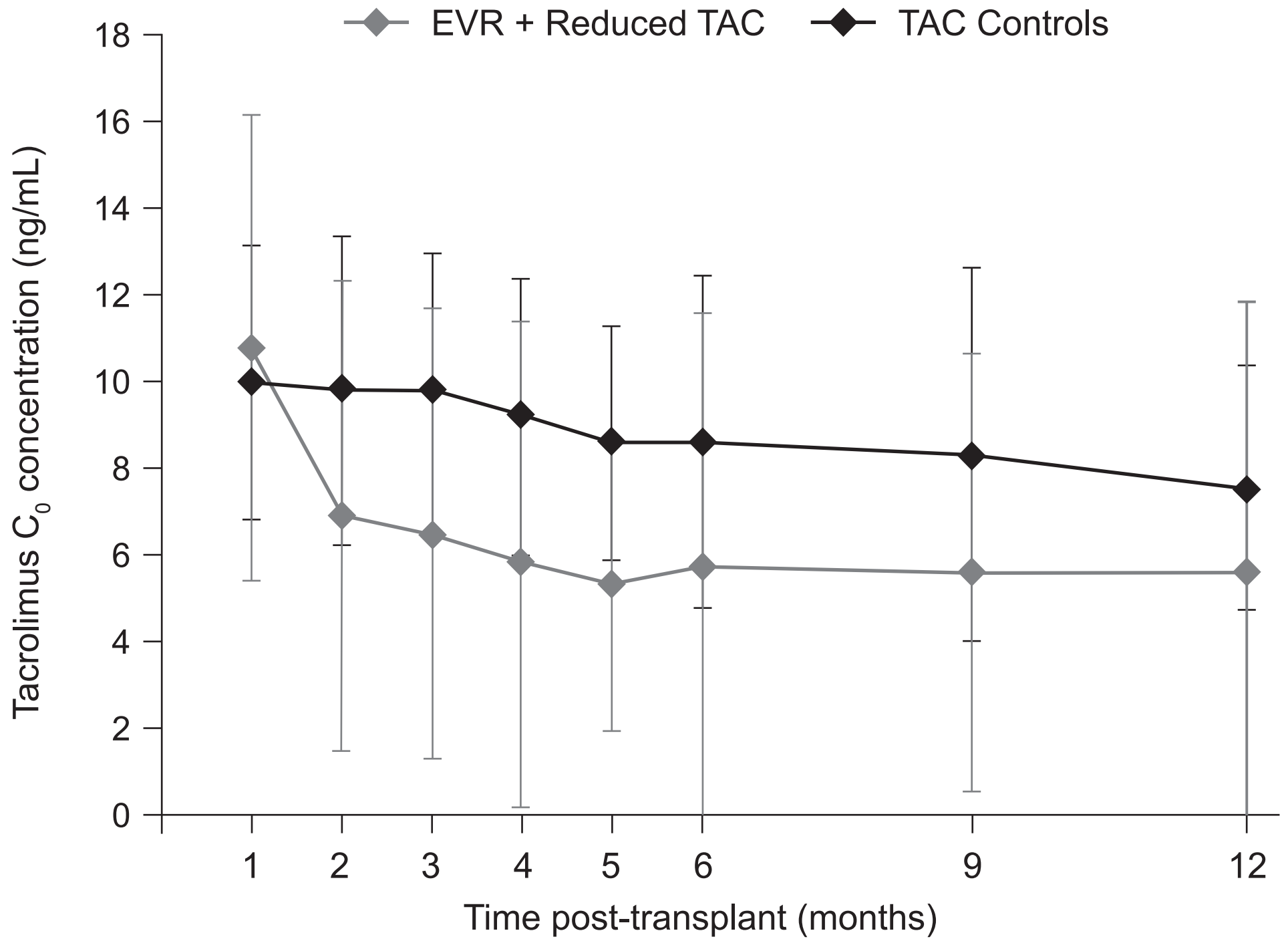

Supplement: Supplementary file 2 [file ajt0012-3008-SD2.pdf]
